# Supplementary material for: Sodium butyrate is incorporated into central metabolism in fly head while inducing oxygen consumption increase
Source: PLoS One. 2024 Dec 19;19(12):e0315892. doi: 10.1371/journal.pone.0315892 (PMC11658510; doi:10.1371/journal.pone.0315892)
Supplement: S1 File — (DOCX) [file pone.0315892.s001.docx]

Sodium butyrate is incorporated into central metabolism in fly head while inducing oxygen consumption increase

**Authors:** Annika Müller-Eigner^1^, Benedikt Gille^1^, Frederik Dethloff^2^, Chen Meng^3^, Christina Ludwig^3^, John T Heiker^4^, Patrick Giavalisco^2^ and Shahaf Peleg^1^*

**Affiliations:**

^1^Research Group Energy Metabolism and Epigenetics, Research Institute for Farm Animal Biology (FBN), 18196 Dummerstorf, Germany

^2^Max Planck Institute for Biology of Ageing, Cologne, Germany

^3^Bavarian Center for Biomolecular Mass Spectrometry (BayBioMS), School of Life Sciences, Technical University of Munich, 85354, Freising, Germany

^4^Helmholtz Institute for Metabolic, Obesity and Vascular Research (HI-MAG) of the Helmholtz Zentrum München at the University of Leipzig and University Hospital Leipzig, Leipzig, Germany

*Correspondence:

peleg@fbn-dummerstorf.de

**Supporting Information**

Supplementary Figures:


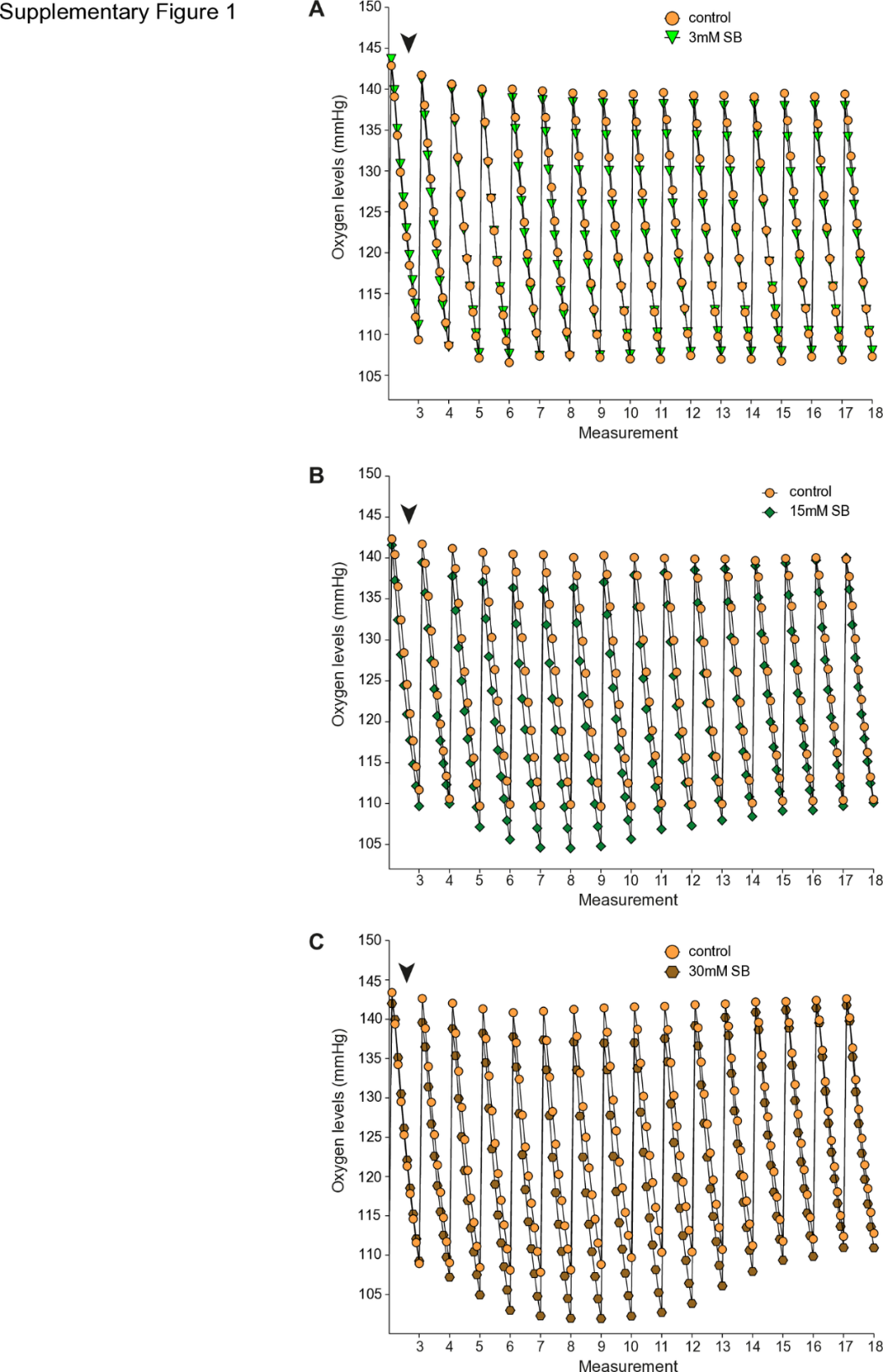


**Supplementary Figure 1: Representative oxygen levels upon the addition of Sodium Butyrate (SB)**

Representative oxygen level changes in control and 3mM SB-treated fly heads (A), control and 15mM SB-treated fly heads (B), control and 30mM SB-treated fly heads (C). Each measurement lasted 2 min and consisted of 10 sub-measurements (ticks). The arrowhead indicates the addition of SB or buffer (control). The last measurement before (3) and all measurements after injection (4-18) are shown.


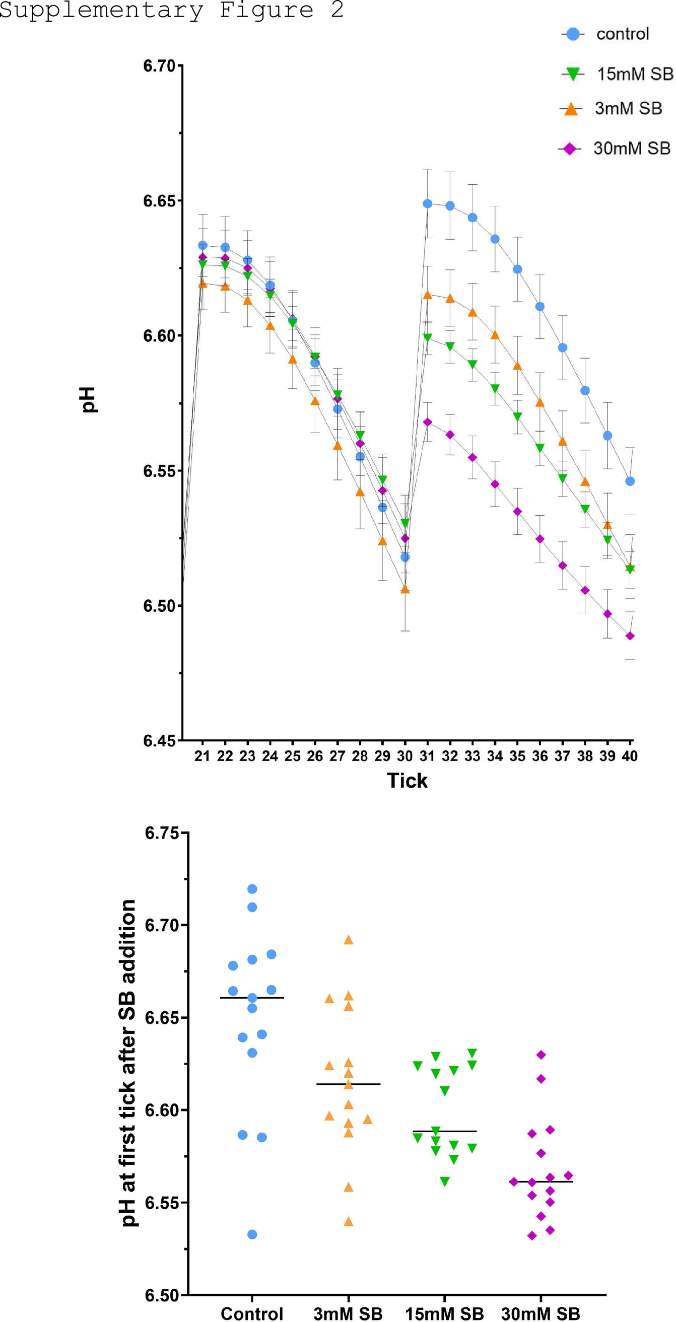


**Supplementary Figure 2: pH analysis following the addition of SB**

(A) Changes in pH during the measurements before and after the addition of SB. The addition of SB causes slight acidification of the media in a concentration dependent manner. (B) Comparison of the pH levels of the first tick between control 3mM, 15mM and 30Mm. Detailed statistical analysis was performed using one-way ANOVA, Tukey´s post hoc test. Control vs 3mM adj pVal = 0.072, control vs 15mM adj pVal = 0.0027, control vs 30mM adj pVal < 0.001, 3mM vs 15mM adj pVal = 0.63, 3mM vs 30mM adj pVal = 0.0049, 15mM vs 30mM adj pVal = 0.11. Error bars indicate ±S.E.M.


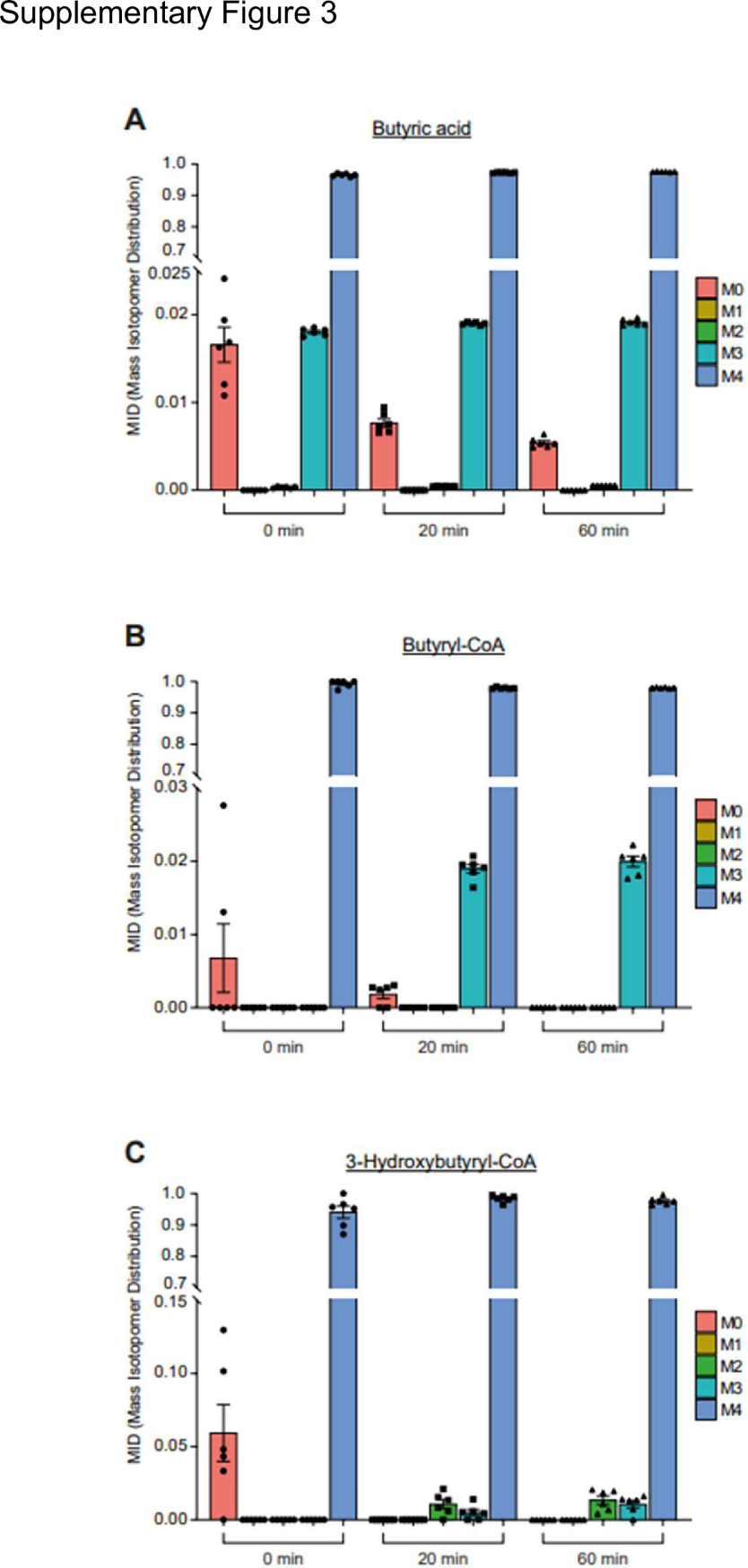


**Supplementary Figure 3: Isotopomer Distribution of butyric derivatives**

Analysis of Mass Isotopomer Distribution (MID) of butyric acid (A), butyryl-CoA (B) and 3-hydroxybutyryl-CoA (C) following ^13^C_4_-SB treatment to determine its incorporation at different time points by mass spectrometry. Error bars indicate ±S.E.M in all graphs. n=6 for all measured metabolites and time points. M0 unlabeled mass of isotope, M+n native metabolite mass + number of isotopically labeled carbons. Detailed statistical analysis was performed using one-way ANOVA, Tukey´s post hoc test, results are presented in Supplementary Table 7.


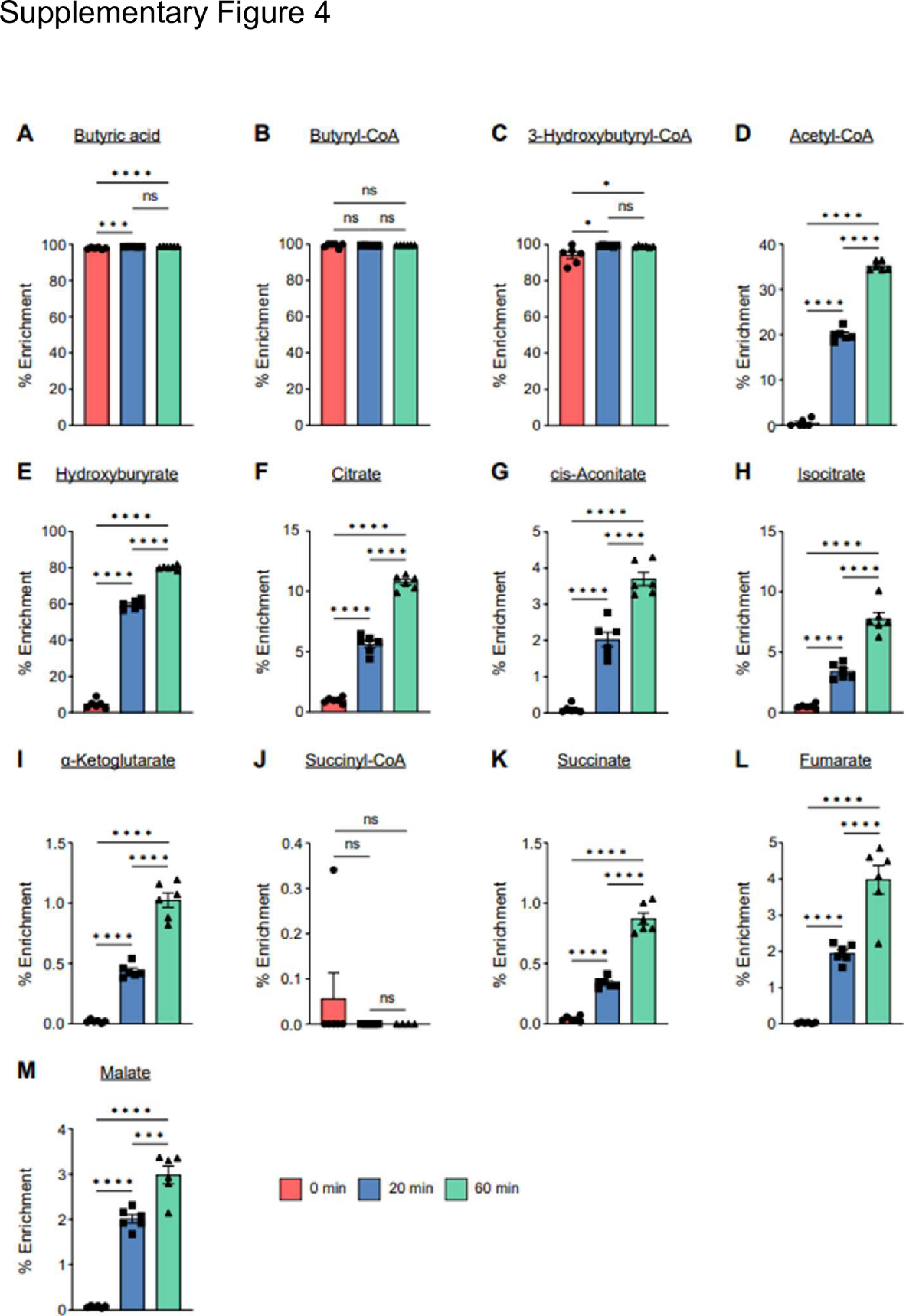


**Supplementary Figure 4: Enrichment analysis of key metabolites in response to ^13^C_4_-labeled SB treatment**

(A-M) Enrichment analysis of key metabolites in response to ^13^C_4_-labeled SB treatment in fly heads at different time points (red, 0 min; blue, 20 min; green, 60 min). n=6 for all measured metabolites and time points, except for: succinyl-CoA 60 min n=4. Error bars indicate ±S.E.M in all graphs. One-way ANOVA, Tukey´s post hoc test, ns p>0.05; * p≤0.05; ** p≤0.01; *** p≤0.001; **** p≤0.0001. Detailed statistical information is presented in Supplementary Table 8.


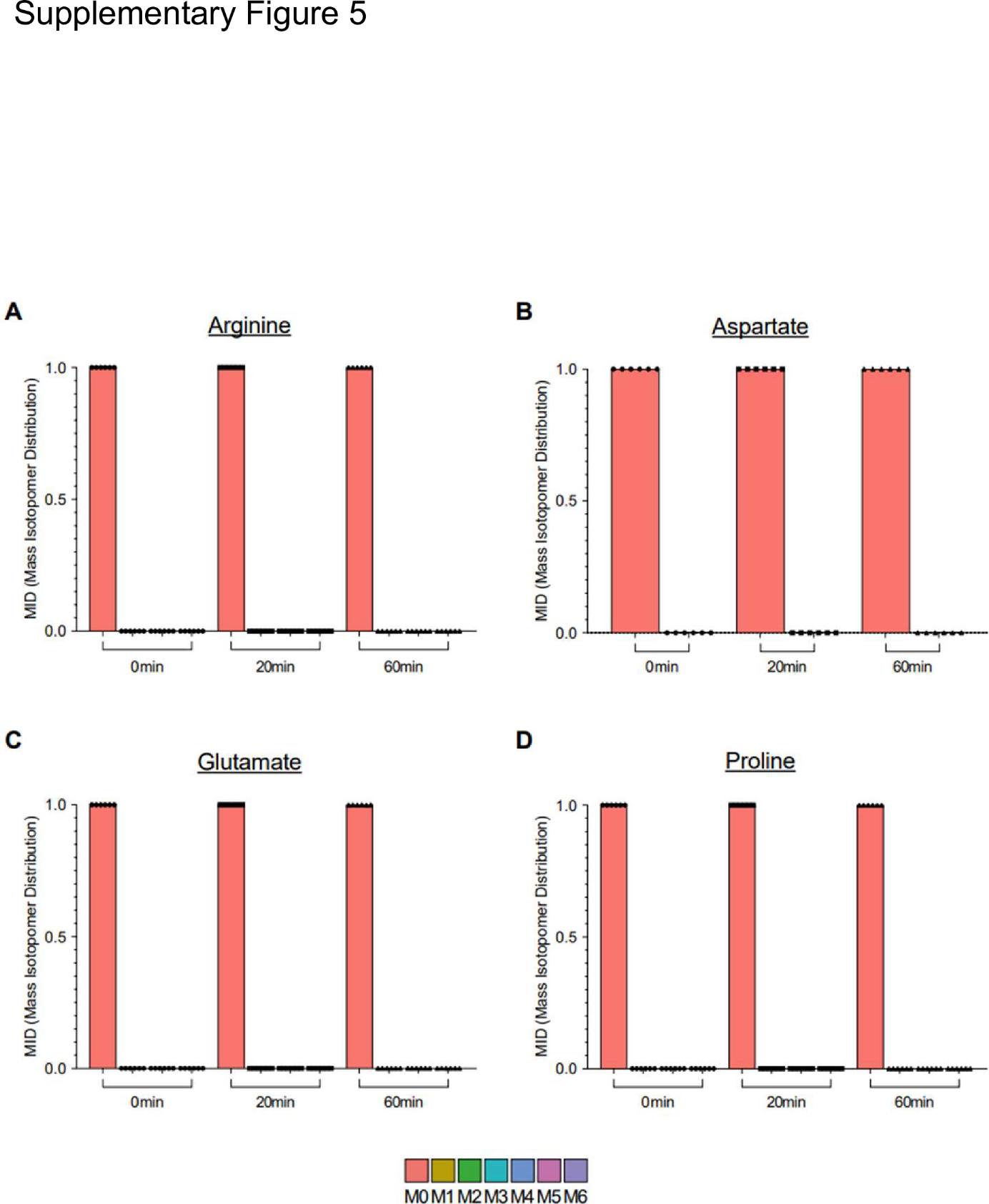


**Supplementary Figure 5: 13C labelling is not detected in amino acids after 20 min of labelled SB treatment**

Analysis of Mass Isotopomer Distribution (MID) of representative metabolites: A) Arginine, B) Aspartate, C) Glutamate, D) Proline. n=6 for measured metabolites and time points. M0 unlabeled mass of isotope, M+n native metabolite mass + number of isotopically labeled carbons.


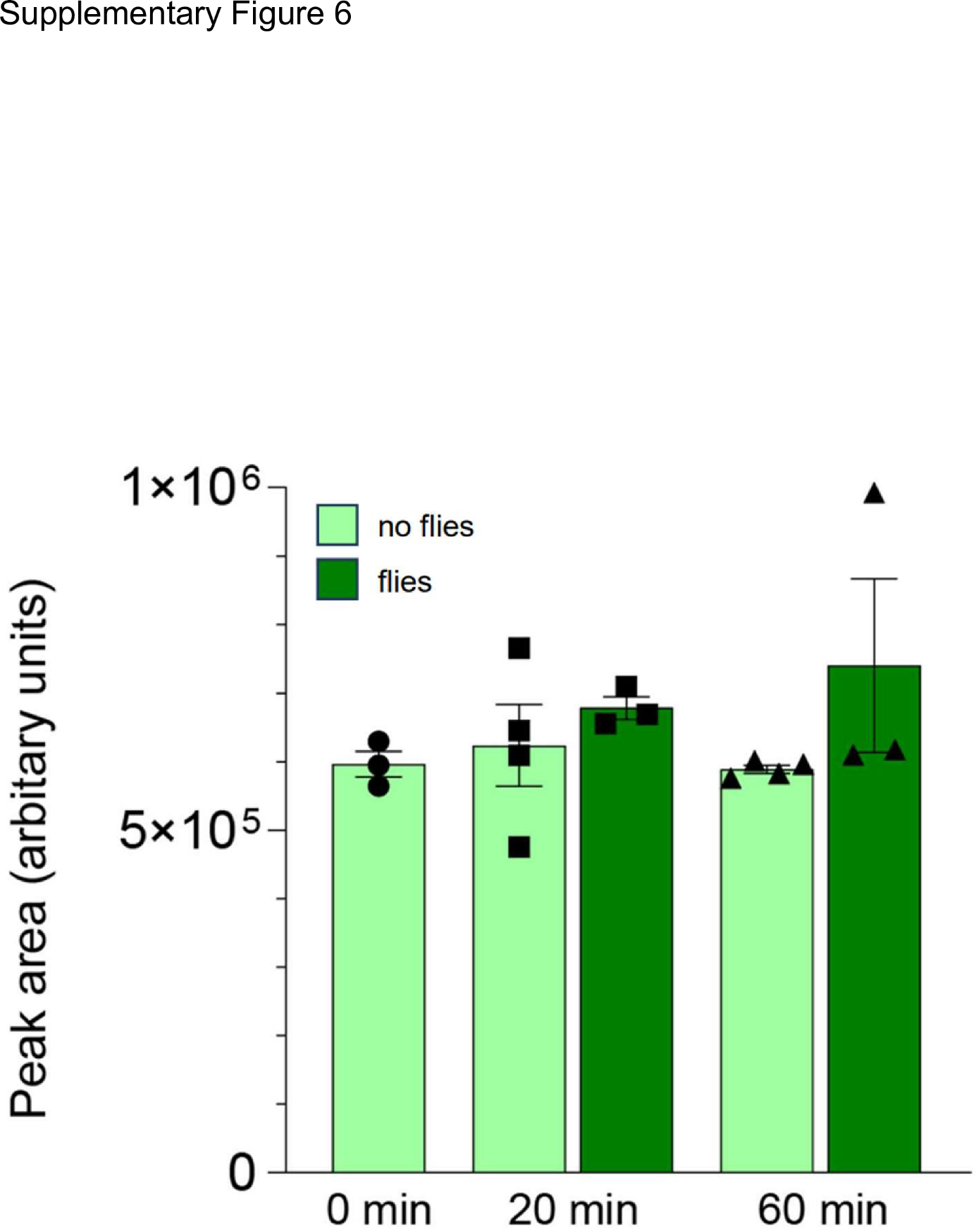


**Supplementary Figure 6: Mass spectrometry analysis of 15mM SB in the supernatant over time**

Supernatant samples were collected at three different time points (0 min, 20 min and 60 min) and analyzed by mass spectrometry for SB. The supernatant was collected from wells without fly heads (light green) and with fly heads (dark green) inside. 0 min, no fly heads n=3; 20 min, no fly heads n=4; 60 min, no fly heads n=4; 20 min, fly heads n=3; 60 min, fly heads n=3. Error bars indicate ±S.E.M in all groups. Two-way ANOVA, Tukey´s post hoc test, ns p>0.05. results are presented in Supplementary Table 9.

**Supplementary Tables**

**Supplementary Table 1: To Figure 1, A**

Two-way ANOVA, Tukey´s multiple comparison test

| **Source of Variation** | **% of total variation** | **P value** |
| --- | --- | --- |
| Interaction | 10.98 | <0.0001 (****) |
| Row Factor (16) | 19.73 | <0.0001 (****) |
| Column Factor (4) | 16.95 | <0.0001 (****) |

| **Time after injection** | **Comparison** | **Adjusted p-value** |
| --- | --- | --- |
| 7.5 min | control – 3 mM SB | 0.0027 (**) |
|  | control – 15 mM SB | 0.9475 (ns) |
|  | control – 30 mM SB | 0.1174 (ns) |
|  | 3 mM SB – 15 mM SB | 0.0008 (***) |
|  | 3 mM SB – 30 mM SB | <0.0001 (****) |
|  | 15 mM SB – 30 mM SB | 0.3456 (ns) |
| 15 min | control – 3 mM SB | 0.8498 (ns) |
|  | control – 15 mM SB | 0.0090 (**) |
|  | control – 30 mM SB | <0.0001 (****) |
|  | 3 mM SB – 15 mM SB | 0.0027 (**) |
|  | 3 mM SB – 30 mM SB | <0.0001 (****) |
|  | 15 mM SB – 30 mM SB | 0.0275 (*) |
| 22.5 min | control – 3 mM SB | 0.8854 (ns) |
|  | control – 15 mM SB | <0.0001 (****) |
|  | control – 30 mM SB | <0.0001 (****) |
|  | 3 mM SB – 15 mM SB | <0.0001 (****) |
|  | 3 mM SB – 30 mM SB | <0.0001 (****) |
|  | 15 mM SB – 30 mM SB | 0.0960 (ns) |
| 30 min | control – 3 mM SB | 0.4134 (ns) |
|  | control – 15 mM SB | <0.0001 (****) |
|  | control – 30 mM SB | <0.0001 (****) |
|  | 3 mM SB – 15 mM SB | <0.0001 (****) |
|  | 3 mM SB – 30 mM SB | <0.0001 (****) |
|  | 15 mM SB – 30 mM SB | 0.0796 (ns) |
| 37.5 min | control – 3 mM SB | 0.2418 (ns) |
|  | control – 15 mM SB | <0.0001 (****) |
|  | control – 30 mM SB | <0.0001 (****) |
|  | 3 mM SB – 15 mM SB | 0.0049 (**) |
|  | 3 mM SB – 30 mM SB | <0.0001 (****) |
|  | 15 mM SB – 30 mM SB | 0.0015 (**) |
| 45 min | control – 3 mM SB | 0.4769 (ns) |
|  | control – 15 mM SB | 0.0021 (**) |
|  | control – 30 mM SB | <0.0001 (****) |
|  | 3 mM SB – 15 mM SB | 0.3117 (ns) |
|  | 3 mM SB – 30 mM SB | <0.0001 (****) |
|  | 15 mM SB – 30 mM SB | <0.0001 (****) |
| 52.5 min | control – 3 mM SB | 0.6300 (ns) |
|  | control – 15 mM SB | 0.1123 (ns) |
|  | control – 30 mM SB | <0.0001 (****) |
|  | 3 mM SB – 15 mM SB | 0.8520 (ns) |
|  | 3 mM SB – 30 mM SB | <0.0001 (****) |
|  | 15 mM SB – 30 mM SB | <0.0001 (****) |

| 60 min | control – 3 mM SB | 0.4105 (ns) |
| --- | --- | --- |
|  | control – 15 mM SB | 0.1683 (ns) |
|  | control – 30 mM SB | <0.0001 (****) |
|  | 3 mM SB – 15 mM SB | 0.9898 (ns) |
|  | 3 mM SB – 30 mM SB | <0.0001 (****) |
|  | 15 mM SB – 30 mM SB | <0.0001 (****) |
| 67.5 min | control – 3 mM SB | 0.4151 (ns) |
|  | control – 15 mM SB | 0.2052 (ns) |
|  | control – 30 mM SB | <0.0001 (****) |
|  | 3 mM SB – 15 mM SB | 0.9957 (ns) |
|  | 3 mM SB – 30 mM SB | 0.0110 (*) |
|  | 15 mM SB – 30 mM SB | 0.0110 (*) |
| 75 min | control – 3 mM SB | 0.2567 (ns) |
|  | control – 15 mM SB | 0.1227 (ns) |
|  | control – 30 mM SB | 0.0019 (**) |
|  | 3 mM SB – 15 mM SB | 0.9986 (ns) |
|  | 3 mM SB – 30 mM SB | 0.3816 (ns) |
|  | 15 mM SB – 30 mM SB | 0.4045 (ns) |
| 82.5 min | control – 3 mM SB | 0.2525 (ns) |
|  | control – 15 mM SB | 0.0716 (ns) |
|  | control – 30 mM SB | 0.0755 (ns) |
|  | 3 mM SB – 15 mM SB | 0.9854 (ns) |
|  | 3 mM SB – 30 mM SB | 0.9500 (ns) |
|  | 15 mM SB – 30 mM SB | 0.9955 (ns) |
| 90 min | control – 3 mM SB | 0.2210 (ns) |
|  | control – 15 mM SB | 0.0639 (ns) |
|  | control – 30 mM SB | 0.2718 (ns) |
|  | 3 mM SB – 15 mM SB | 0.9887 (ns) |
|  | 3 mM SB – 30 mM SB | 0.9999 (ns) |
|  | 15 mM SB – 30 mM SB | 0.9807 (ns) |
| 97.5 min | control – 3 mM SB | 0.2379 (ns) |
|  | control – 15 mM SB | 0.0897 (ns) |
|  | control – 30 mM SB | 0.4974 (ns) |
|  | 3 mM SB – 15 mM SB | 0.9953 (ns) |
|  | 3 mM SB – 30 mM SB | 0.9801 (ns) |
|  | 15 mM SB – 30 mM SB | 0.9156 (ns) |
| 105 min | control – 3 mM SB | 0.1310 (ns) |
|  | control – 15 mM SB | 0.0752 (ns) |
|  | control – 30 mM SB | 0.3786 (ns) |
|  | 3 mM SB – 15 mM SB | >0.9999 (ns) |
|  | 3 mM SB – 30 mM SB | 0.9655 (ns) |
|  | 15 mM SB – 30 mM SB | 0.9535 (ns) |
| 112.5 min | control – 3 mM SB | 0.2061 (ns) |
|  | control – 15 mM SB | 0.0609 (ns) |
|  | control – 30 mM SB | 0.7840 (ns) |
|  | 3 mM SB – 15 mM SB | 0.9905 (ns) |
|  | 3 mM SB – 30 mM SB | 0.8212 (ns) |
|  | 15 mM SB – 30 mM SB | 0.6096 (ns) |

**Supplementary Table 2: To Figure 1, B, C:**

| **Figure** | **Condition** | **Mean ± SEM** | | **n** | **CI 95% of mean** |
| --- | --- | --- | --- | --- | --- |
| 1, B | control | -1.413 ± 0.3346 | | 28 | -2.099 – -0.7260 |
|  | 3 mM SB | -1.062 ± 0.3598 | | 16 | -1.829 – -0.2954 |
|  | 15 mM SB | 2.066 ± 0.8069 | | 22 | 0.3875 – 3.744 |
|  | 30 mM SB | 4.463 ± 0.8803 | | 15 | 2.575 – 6.352 |
| 1, C | control | -2.830 ± 0.4356 | | 28 | -3.724 – -1.936 |
|  | 3 mM SB | -1.647 ± 0.2967 | | 16 | -2.279 – -1.015 |
|  | 15 mM SB | -1.241 ± 0.3909 | | 22 | -2.054 – -0.4280 |
|  | 30 mM SB | 1.671 ± 0.3987 | | 15 | 0.8161 – 2.526 |
| **Figure** | **Comparison** | | **Adjusted p-value** | **One-way ANOVA, Tukey´s multiple comparison test** | |
| 1, B | control – 3 mM SB | | 0.9770 (ns) | P value < 0.0001; R squared 0.4250 | |
|  | control – 15 mM SB | | 0.0002 (***) |  |  |
|  | control – 30 mM SB | | < 0.0001 (****) |  |  |
|  | 3 mM SB – 15 mM SB | | 0.0046 (**) |  |  |
|  | 3 mM SB – 30 mM SB | | < 0.0001 (****) |  |  |
|  | 15 mM SB – 30 mM SB | | 0.0519 (ns) |  |  |
| 1, C | control – 3 mM SB | | 0.1892 (ns) | P value < 0.0001; R squared 0.4267 | |
|  | control – 15 mM SB | | 0.0194 (*) |  |  |
|  | control – 30 mM SB | | < 0.0001 (****) |  |  |
|  | 3 mM SB – 15 mM SB | | 0.9111 (ns) |  |  |
|  | 3 mM SB – 30 mM SB | | < 0.0001 (****) |  |  |
|  | 15 mM SB – 30 mM SB | | < 0.0001 (****) |  |  |

**Supplementary Table 3: To Supplementary Figure 2, 3**

**Proteome**

| **Protein ID** | **CG number** | **Control** | **Mean ± SEM** | **SB** | **Mean ± SEM** | **Student t-test adjusted p-Value** |
| --- | --- | --- | --- | --- | --- | --- |
| P91929  (NADH dehydrogenase [ubiquinone] 1 alpha subcomplex subunit 10) | CG6343 (ND-42) | 9.4224 | 9.3960 ± 0.0223 | 9.3791 | 9.4083 ± 0.0113 | 0.9698 |
|  |  | 9.3975 |  | 9.4088 |  |  |
|  |  | 9.3329 |  | 9.4344 |  |  |
|  |  | 9.4320 |  | 9.4111 |  |  |
| Q94511  (NADH-ubiquinone oxidoreductase 75 kDa subunit) | CG2286 (ND-75) | 9.8504 | 9.8633 ± 0.0082 | 9.8714 | 9.8756 ± 0.0132 | 0.9449 |
|  |  | 9.8497 |  | 9.8457 |  |  |
|  |  | 9.8838 |  | 9.9099 |  |  |
|  |  | 9.8691 |  | 9.8752 |  |  |
| Q94523  (Succinate dehydrogenase [ubiquinone] flavoprotein subunit) | CG17246 (SdhA) | 10.0389 | 10.0286 ±  0.0064 | 10.0535 | 10.0404 ±  0.0070 | 0.9449 |
|  |  | 10.0341 |  | 10.0207 |  |  |
|  |  | 10.0099 |  | 10.0415 |  |  |
|  |  | 10.0316 |  | 10.0457 |  |  |
| Q9VGS3  (Succinate dehydrogenase cytochrome b560 subunit) | CG6666 (SdhC) | 8.7732 | 8.8062 ±  0.0155 | 8.7039 | 8.8031 ±  0.0396 | 0.9983 |
|  |  | 8.8249 |  | 8.7884 |  |  |
|  |  | 8.8391 |  | 8.8264 |  |  |
|  |  | 8.7876 |  | 8.8939 |  |  |

| Q9XY35  (Cytochrome b-c1 complex subunit 9) | CG8764 (ox) | 9.1050 | 9.0880 ± 0.0184 | 9.0835 | 9.1133 ± 0.0152 | 0.9449 |
| --- | --- | --- | --- | --- | --- | --- |
|  |  | 9.1014 |  | 9.1305 |  |  |
|  |  | 9.0334 |  | 9.0922 |  |  |
|  |  | 9.1123 |  | 9.1472 |  |  |
| Q9VQ29  (Cytochrome b-c1 complex subunit Rieske) | CG7361 (RFeSP) | 9.6808 | 9.6663 ± 0.0105 | 9.6726 | 9.6621 ± 0.0143 | 0.9774 |
|  |  | 9.6350 |  | 9.6784 |  |  |
|  |  | 9.6762 |  | 9.6193 |  |  |
|  |  | 9.6730 |  | 9.6782 |  |  |
| Q94514  (Cytochrome c oxidase subunit 5A) | CG14724 (COX5A) | 9.9490 | 9.9225 ± 0.0202 | 9.9412 | 9.9427 ± 0.0054 | 0.9449 |
|  |  | 9.9320 |  | 9.9532 |  |  |
|  |  | 9.8628 |  | 9.9482 |  |  |
|  |  | 9.9461 |  | 9.9281 |  |  |
| Q9VHS2 (Cytochrome c oxidase subunit 7A | CG9603 (COX7A) | 9.4119 | 9.4116 ± 0.0069 | 9.4161 | 9.4198 ± 0.0027 | 0.9449 |
|  |  | 9.3974 |  | 9.4171 |  |  |
|  |  | 9.4069 |  | 9.4179 |  |  |
|  |  | 9.4301 |  | 9.4279 |  |  |
| P35381 (ATP synthase subunit alpha) | CG3612 (blw) | 11.0295 | 11.0238 ± 0.0028 | 11.0406 | 11.0249 ± 0.0085 | 0.9956 |
|  |  | 11.0196 |  | 11.0227 |  |  |
|  |  | 11.0278 |  | 11.0343 |  |  |
|  |  | 11.0184 |  | 11.0019 |  |  |
| Q24251 (ATP synthase subunit d) | CG6030 (ATPsyn-d) | 9.9758 | 9.9332 ± 0.0244 | 9.9362 | 9.9442 ± 0.0080 | 0.9713 |
|  |  | 9.9355 |  | 9.9681 |  |  |
|  |  | 9.8642 |  | 9.9364 |  |  |
|  |  | 9.9573 |  | 9.9362 |  |  |
| Q9W401 (Probable citrate synthase) | CG3861 (kdn) | 10.5849 | 10.5707 ± 0.0052 | 10.5991 | 10.5810 ± 0.0074 | 0.9449 |
|  |  | 10.5688 |  | 10.5653 |  |  |
|  |  | 10.5688 |  | 10.5864 |  |  |
|  |  | 10.5601 |  | 10.5734 |  |  |
| Q9VIE8  (Aconitate hydratase) | CG9244 (mAcon1) | 10.6498 | 10.6520 ± 0.0076 | 10.6935 | 10.6662 ± 0.0095 | 0.9449 |
|  |  | 10.6729 |  | 10.6624 |  |  |
|  |  | 10.6488 |  | 10.6590 |  |  |
|  |  | 10.6365 |  | 10.6497 |  |  |
| Q7KUB1  (Isocitrate dehydrogenase [NADP]) | CG7176 (Idh) | 10.3528 | 10.3589 ± 0.0040 | 10.3557 | 10.3568 ± 0.0037 | 0.9713 |
|  |  | 10.3532 |  | 10.3574 |  |  |
|  |  | 10.3599 |  | 10.3479 |  |  |
|  |  | 10.3697 |  | 10.3661 |  |  |
| Q9VA02  (Probable 2-oxoglutarate dehydrogenase E1 component DHKTD1 homolog) | CG1544 | 8.8059 | 8.8110 ± 0.0085 | 8.8028 | 8.8151 ± 0.0094 | 0.9759 |
|  |  | 8.8036 |  | 8.7956 |  |  |
|  |  | 8.7985 |  | 8.8289 |  |  |
|  |  | 8.8361 |  | 8.8332 |  |  |
| Q9VGQ1 (Dihydrolipoyllysine-residue succinyltransferase component of 2-oxoglutarate dehydrogenase complex) | CG5214 | 9.7823 | 9.7797 ± 0.0009 | 9.7817 | 9.7888 ± 0.0060 | 0.9449 |
|  |  | 9.7791 |  | 9.7887 |  |  |
|  |  | 9.7785 |  | 9.8057 |  |  |
|  |  | 9.7788 |  | 9.7792 |  |  |
| Q9VVL7 (Dihydrolipoyl dehydrogenase) | CG7430 | 10.0387 | 10.0342 ± 0.0029 | 10.0382 | 10.0384 ± 0.0096 | 0.9713 |
|  |  | 10.0289 |  | 10.0391 |  |  |
|  |  | 10.0398 |  | 10.0147 |  |  |
|  |  | 10.0293 |  | 10.0616 |  |  |
| Q95U38  (Succinate--CoA ligase [ADP-forming] subunit beta) | CG11963 (skap) | 10.2413 | 10.2056 ± 0.0197 | 10.2254 | 10.2150 ± 0.0050 | 0.9713 |
|  |  | 10.2022 |  | 10.2132 |  |  |
|  |  | 10.1518 |  | 10.2020 |  |  |
|  |  | 10.2271 |  | 10.2196 |  |  |
| Q8IRQ5  (fumarate hydratase) | CG4094 (Fum1) | 9.8735 | 9.8947 ± 0.0158 | 9.8645 | 9.8849 ± 0.0108 | 0.9675 |
|  |  | 9.8653 |  | 9.8808 |  |  |
|  |  | 9.9345 |  | 9.9155 |  |  |
|  |  | 9.9054 |  | 9.8789 |  |  |
| Q9VKX2  (Malate dehydrogenase) | CG5362 (Mdh1) | 10.0215 | 10.0224 ± 0.0050 | 10.0325 | 10.0263 ± 0.0068 | 0.9698 |
|  |  | 10.0261 |  | 10.0249 |  |  |
|  |  | 10.0092 |  | 10.0080 |  |  |
|  |  | 10.0327 |  | 10.0400 |  |  |

**Supplementary Table 4: To Figure 3, B**

Two-way ANOVA, Tukey´s multiple comparison test

| **Source of Variation** | **% of total variation** | **P value** |
| --- | --- | --- |
| Interaction | 5.057 | 0.3102 (ns) |
| Row Factor (16) | 9.491 | <0.0001 (****) |
| Column Factor (3) | 14.68 | <0.0001 (****) |

| **Time after injection** | **Comparison** | **Adjusted p-value** |
| --- | --- | --- |
| 7.5 min | control – 5 mM PP | 0.7662 (ns) |
|  | control – 25 mM PP | 0.0524 (ns) |
|  | 5 mM PP – 25 mM PP | 0.1515 (ns) |
| 15 min | control – 5 mM PP | 0.1123 (ns) |
|  | control – 25 mM PP | 0.0032 (**) |
|  | 5 mM PP – 25 mM PP | 0.1964 (ns) |
| 22.5 min | control – 5 mM PP | 0.0486 (*) |
|  | control – 25 mM PP | 0.0006 (***) |
|  | 5 mM PP – 25 mM PP | 0.1358 (ns) |
| 30 min | control – 5 mM PP | 0.2282 (ns) |
|  | control – 25 mM PP | 0.0001 (***) |
|  | 5 mM PP – 25 mM PP | 0.0089 (**) |
| 37.5 min | control – 5 mM PP | 0.4050 (ns) |
|  | control – 25 mM PP | 0.0002 (***) |
|  | 5 mM PP – 25 mM PP | 0.0050 (**) |
| 45 min | control – 5 mM PP | 0.4655 (ns) |
|  | control – 25 mM PP | 0.0003 (***) |
|  | 5 mM PP – 25 mM PP | 0.0047 (**) |
| 52.5 min | control – 5 mM PP | 0.4269 (ns) |
|  | control – 25 mM PP | 0.0003 (***) |
|  | 5 mM PP – 25 mM PP | 0.0061 (**) |
| 60 min | control – 5 mM PP | 0.4785 (ns) |
|  | control – 25 mM PP | 0.0013 (**) |
|  | 5 mM PP – 25 mM PP | 0.0185 (*) |
| 67.5 min | control – 5 mM PP | 0.4867 (ns) |
|  | control – 25 mM PP | 0.0137 (*) |
|  | 5 mM PP – 25 mM PP | 0.1159 (ns) |
| 75 min | control – 5 mM PP | 0.5303 (ns) |
|  | control – 25 mM PP | 0.1061 (ns) |
|  | 5 mM PP – 25 mM PP | 0.4464 (ns) |
| 82.5 min | control – 5 mM PP | 0.5928 (ns) |
|  | control – 25 mM PP | 0.2190 (ns) |
|  | 5 mM PP – 25 mM PP | 0.6312 (ns) |
| 90 min | control – 5 mM PP | 0.5929 (ns) |
|  | control – 25 mM PP | 0.3860 (ns) |
|  | 5 mM PP – 25 mM PP | 0.8486 (ns) |
| 97.5 min | control – 5 mM PP | 0.7765 (ns) |
|  | control – 25 mM PP | 0.6859 (ns) |
|  | 5 mM PP – 25 mM PP | 0.9558 (ns) |
| 105 min | control – 5 mM PP | 0.5832 (ns) |
|  | control – 25 mM PP | 0.6280 (ns) |
|  | 5 mM PP – 25 mM PP | 0.9930 (ns) |
| 112.5 min | control – 5 mM PP | 0.5671 (ns) |
|  | control – 25 mM PP | 0.6668 (ns) |
|  | 5 mM PP – 25 mM PP | 0.9996 (ns) |

**Supplementary Table 5: To Figure 3, C, D**

| **Figure** | **Condition** | **Mean ± SEM** | | **n** | | **CI 95% of mean** |
| --- | --- | --- | --- | --- | --- | --- |
| 3, C | control | 0.4647 ± 0.8318 | | 11 | | -1.389 – 2.318 |
|  | 5 mM PP | 3.240 ± 1.357 | | 15 | | 0.3300 – 6.150 |
|  | 25 mM PP | 8.008 ± 2.335 | | 7 | | 2.295 – 13.72 |
| 3, D | control | -0.5767 ± 0.9994 | | 11 | | -2.803 – 1.650 |
|  | 5 mM PP | 1.346 ± 0.9356 | | 15 | | -0.6612 – 3.352 |
|  | 25 mM PP | 5.639 ± 1.699 | | 7 | | 1.482 – 9.795 |
| **Figure** | **Comparison** | | **Adjusted p-value** | | **One-way ANOVA, Tukey´s multiple comparison test** | |
| 3, C | control – 5 mM PP | | 0.3452 (ns) | | P value 0.0438; R squared 0.1882 | |
|  | control – 25 mM PP | | 0.0346 (*) | |  |  |
|  | 5 mM PP – 25 mM PP | | 0.2831 (ns) | |  |  |
| 3, D | control – 5 mM PP | | 0.3751 (ns) | | P value 0.0028; R squared 0.3235 | |
|  | control – 25 mM PP | | 0.0021 (**) | |  |  |
|  | 5 mM PP – 25 mM PP | | 0.0239 (*) | |  |  |

**Supplementary Table 6: To Figure 4**

Metabolome - Pool size

| **Metabolite** | | **Time point** | **Mean ± SEM** | | | **n** | **CI 95% of mean** |
| --- | --- | --- | --- | --- | --- | --- | --- |
| Butyric acid | | 0 min | 9.78e+08 ± 1.53e+08 | | | 6 | 5.86e+08 – 1.37e+09 |
|  |  | 20 min | 2.52e+09 ± 1.15e+08 | | | 6 | 2.22e+09 – 2.28e+09 |
|  |  | 60 min | 4.27e+09 ± 1.35e+08 | | | 6 | 3.93e+09 – 4.62e+09 |
| Butyryl-CoA | | 0 min | 4.05e+05 ± 2.70e+04 | | | 6 | 3.36e+05 – 4.75e+05 |
|  |  | 20 min | 2.79e+06 ± 1.14e+05 | | | 6 | 2.50e+06 – 3.09e+06 |
|  |  | 60 min | 3.13e+06 ± 1.06e+05 | | | 6 | 2.86e+06 – 3.41e+06 |
| 3-Hydroxybutyryl-CoA | | 0 min | 1.42e+05 ± 9.78e+03 | | | 6 | 1.17e+05 – 1.67e+05 |
|  |  | 20 min | 5.35e+05 ± 3.42e+04 | | | 6 | 4.47e+05 – 6.23e+05 |
|  |  | 60 min | 7.11e+05 ± 3.45e+04 | | | 6 | 6.22e+05 – 7.99e+05 |
| **Metabolite** | **Comparison** | | | **Adjusted p-value** | **One-wayANOVA, Tukey´s multiple comparison test** | | |
| Butyric acid | 0 min – 20 min | | | < 0.0001 (****) | P value < 0.0001; R squared 0.9520 | | |
|  | 0 min – 60 min | | | < 0.0001 (****) |  |  |  |
|  | 20 min – 60 min | | | < 0.0001 (****) |  |  |  |
| Butyryl-CoA | 0 min – 20 min | | | < 0.0001 (****) | P value < 0.0001; R squared 0.9724 | | |
|  | 0 min – 60 min | | | < 0.0001 (****) |  |  |  |
|  | 20 min – 60 min | | | 0.0467 (*) |  |  |  |
| 3-Hydroxybutyryl-CoA | 0 min – 20 min | | | < 0.0001 (****) | P value < 0.0001; R squared 0.9325 | | |
|  | 0 min – 60 min | | | < 0.0001 (****) |  |  |  |
|  | 20 min – 60 min | | | 0.0016 (**) |  |  |  |

**Supplementary Table 7: To Figure 5 and Supplementary Figure 4, 6**

Metabolome - MID

| **Metabolite (Isotopomer)** | | **Time point** | **Mean ± SEM** | | | **n** | **CI 95% of mean** |
| --- | --- | --- | --- | --- | --- | --- | --- |
| Butyric acid (M4) | | 0 min | 0.9651 ± 0.001829 | | | 6 | 0.9604 – 0.9698 |
|  |  | 20 min | 0.9729 ± 0.0004276 | | | 6 | 0.9718 – 0.9740 |
|  |  | 60 min | 0.9749 ± 0.0002052 | | | 6 | 0.9744 – 0.9755 |
| Butyryl-CoA (M4) | | 0 min | 0.9932 ± 0.004686 | | | 6 | 0.9812 – 1.005 |
|  |  | 20 min | 0.9792 ± 0.001056 | | | 6 | 0.9765 – 09819 |
|  |  | 60 min | 0.9801 ± 0.0007087 | | | 6 | 0.9782 – 0.9819 |
| 3-Hydroxybutyryl-CoA (M4) | | 0 min | 0.9407 ± 0.01941 | | | 6 | 0.8908 – 0.9906 |
|  |  | 20 min | 0.9844 ± 0.004381 | | | 6 | 0.9732 – 0.9957 |
|  |  | 60 min | 0.9760 ± 0.004688 | | | 6 | 0.9640 – 0.9881 |
| Acetyl-CoA (M2) | | 0 min | 0.004816 ± 0.003199 | | | 6 | -0.003406 – 0.01304 |
|  |  | 20 min | 0.1998 ± 0.005458 | | | 6 | 0.1858 – 0.2138 |
|  |  | 60 min | 0.3517 ± 0.004083 | | | 6 | 0.3412 – 0.3622 |
| Hydroxybutyrate (M4) | | 0 min | 0.04576 ± 0.009993 | | | 6 | 0.02007 – 0.07145 |
|  |  | 20 min | 0.5902 ± 0.01003 | | | 6 | 0.5644 – 0.6159 |
|  |  | 60 min | 0.7883 ± 0.004394 | | | 6 | 0.7770 – 0.7996 |
| Citrate (M2) | | 0 min | 0.02629 ± 0.002579 | | | 6 | 0.01966 – 0.03292 |
|  |  | 20 min | 0.1230 ± 0.007035 | | | 6 | 0.1049 – 0.1411 |
|  |  | 60 min | 0.1929 ± 0.003195 | | | 6 | 0.1847 – 0.2011 |
| cis-Aconitate (M2) | | 0 min | 0.003246 ± 0.001231 | | | 6 | -0.0001766 – 0.006668 |
|  |  | 20 min | 0.05973 ± 0.005309 | | | 6 | 0.04608 – 0.07338 |
|  |  | 60 min | 0.1069 ± 0.005041 | | | 6 | 0.09392 – 0.1198 |
| Isocitrate (M2) | | 0 min | 0.01581 ± 0.002227 | | | 6 | 0.01009 – 0.02154 |
|  |  | 20 min | 0.09812 ± 0.007365 | | | 6 | 0.07919 – 0.1171 |
|  |  | 60 min | 0.1816 ± 0.009907 | | | 6 | 0.1562 – 0.2071 |
| α-Ketoglutarate (M2) | | 0 min | 0.0005344 ± 0.0001288 | | | 6 | 0.0002034 – 0.0008655 |
|  |  | 20 min | 0.008312 ± 0.0003407 | | | 6 | 0.007436 – 0.009188 |
|  |  | 60 min | 0.01373 ± 0.0006 | | | 6 | 0.01219 – 0.01528 |
| Succinyl-CoA (M2) | | 0 min | 0 ± 0 | | | 6 | 0 – 0 |
|  |  | 20 min | 0 ± 0 | | | 6 | 0 – 0 |
|  |  | 60 min | 0 ± 0 | | | 4 | 0 – 0 |
| Succinate (M2) | | 0 min | 0.0001139 ± 3.580e-005 | | | 6 | 2.186e-005 – 0.0002059 |
|  |  | 20 min | 0.004689 ± 0.0003089 | | | 6 | 0.003895 – 0.005483 |
|  |  | 60 min | 0.01126 ± 0.0005978 | | | 6 | 0.009728 – 0.01280 |
| Fumarate (M2) | | 0 min | 0.0006052 ± 0.0001504 | | | 6 | 0.0002186 – 0.0009919 |
|  |  | 20 min | 0.02859 ± 0.001385 | | | 6 | 0.02503 – 0.03215 |
|  |  | 60 min | 0.04188 ± 0.003370 | | | 6 | 0.03322 – 0.05054 |
| Malate (M2) | | 0 min | 0.001399 ± 0.0001903 | | | 6 | 0.0009102 – 0.001889 |
|  |  | 20 min | 0.02760 ± 0.001196 | | | 6 | 0.02452 – 0.03067 |
|  |  | 60 min | 0.03439 ± 0.001715 | | | 6 | 0.02998 – 0.03879 |
| **Metabolite (Isotopomer)** | **Comparison** | | | **Adjusted p-value** | **One-way ANOVA, Tukey´s multiple comparison test** | | |
| Butyric acid (M4) | 0 min – 20 min | | | 0.0004 (***) | P value < 0.0001; R squared 0.7511 | | |
|  | 0 min – 60 min | | | < 0.0001 (****) |  |  |  |
|  | 20 min – 60 min | | | 0.4090 (ns) |  |  |  |
| Butyryl-CoA (M4) | 0 min – 20 min | | | 0.0079 (**) | P value 0.0046; R squared 0.5118 | | |
|  | 0 min – 60 min | | | 0.0122 (*) |  |  |  |
|  | 20 min – 60 min | | | 0.9738 (ns) |  |  |  |
| 3-Hydroxybutyryl-CoA (M4) | 0 min – 20 min | | | 0.0479 (*) | P value 0.0442; R squared 0.3402 | | |
|  | 0 min – 60 min | | | 0.1197 (ns) |  |  |  |
|  | 20 min – 60 min | | | 0.8714 (ns) |  |  |  |
| Acetyl-CoA (M2) | 0 min – 20 min | | | < 0.0001 (****) | P value < 0.0001; R squared 0.9953 | | |
|  | 0 min – 60 min | | | < 0.0001 (****) |  |  |  |
|  | 20 min – 60 min | | | < 0.0001 (****) |  |  |  |
| Hydroxybutyrate (M4) | 0 min – 20 min | | | < 0.0001 (****) | P value < 0.0001; R squared 0.9963 | | |
|  | 0 min – 60 min | | | < 0.0001 (****) |  |  |  |
|  | 20 min – 60 min | | | < 0.0001 (****) |  |  |  |
| Citrate (M2) | 0 min – 20 min | | | < 0.0001 (****) | P value < 0.0001; R squared 0.9768 | | |
|  | 0 min – 60 min | | | < 0.0001 (****) |  |  |  |
|  | 20 min – 60 min | | | < 0.0001 (****) |  |  |  |
| cis-Aconitate (M2) | 0 min – 20 min | | | < 0.0001 (****) | P value < 0.0001; R squared 0.9511 | | |
|  | 0 min – 60 min | | | < 0.0001 (****) |  |  |  |
|  | 20 min – 60 min | | | < 0.0001 (****) |  |  |  |
| Isocitrate (M2) | 0 min – 20 min | | | < 0.0001 (****) | P value < 0.0001; R squared 0.9459 | | |
|  | 0 min – 60 min | | | < 0.0001 (****) |  |  |  |
|  | 20 min – 60 min | | | < 0.0001 (****) |  |  |  |
| α-Ketoglutarate (M2) | 0 min – 20 min | | | < 0.0001 (****) | P value < 0.0001; R squared 0.9728 | | |
|  | 0 min – 60 min | | | < 0.0001 (****) |  |  |  |
|  | 20 min – 60 min | | | < 0.0001 (****) |  |  |  |
| Succinyl-CoA (M2) | 0 min – 20 min | | | no data for this isotopomer was measured, no analysis possible | | | |
|  | 0 min – 60 min | | |  |  |  |  |
|  | 20 min – 60 min | | |  |  |  |  |
| Succinate (M2) | 0 min – 20 min | | | < 0.0001 (****) | P value < 0.0001; R squared 0.9651 | | |
|  | 0 min – 60 min | | | < 0.0001 (****) |  |  |  |
|  | 20 min – 60 min | | | < 0.0001 (****) |  |  |  |
| Fumarate (M2) | 0 min – 20 min | | | < 0.0001 (****) | P value < 0.0001; R squared 0.9303 | | |
|  | 0 min – 60 min | | | < 0.0001 (****) |  |  |  |
|  | 20 min – 60 min | | | 0.0012 (**) |  |  |  |
| Malate (M2) | 0 min – 20 min | | | < 0.0001 (****) | P value < 0.0001; R squared 0.9650 | | |
|  | 0 min – 60 min | | | < 0.0001 (****) |  |  |  |
|  | 20 min – 60 min | | | 0.0034 (**) |  |  |  |

**Supplementary Table 8: To Supplementary Figure 4**

Metabolome - Enrichment

| **Metabolite** | **Time point** | **Mean ± SEM** | **n** | **CI 95% of mean** |
| --- | --- | --- | --- | --- |
| Butyric acid | 0 min | 97.88 ± 0.1939 | 6 | 97.38 – 98.38 |
|  | 20 min | 98.74 ± 0.04604 | 6 | 98.62 – 98.85 |
|  | 60 min | 98.95 ± 0.02124 | 6 | 98.90 – 99.01 |
| Butyryl-CoA | 0 min | 99.32 ± 0.4686 | 6 | 98.12 – 100.5 |
|  | 20 min | 99.34 ± 0.06858 | 6 | 99.17 – 99.52 |
|  | 60 min | 99.50 ± 0.01772 | 6 | 99.46 – 99.55 |
| 3-Hydroxybutyryl-CoA | 0 min | 94.07 ± 1.941 | 6 | 89.08 – 99.06 |
|  | 20 min | 99.34 ± 0.1815 | 6 | 98.88 – 99.81 |
|  | 60 min | 99.07 ± 0.1847 | 6 | 98.59 – 99.54 |
| Acetyl-CoA | 0 min | 0.4816 ± 0.3199 | 6 | -0.3406 – 1.304 |
|  | 20 min | 19.98 ± 0.5458 | 6 | 18.58 – 21.38 |
|  | 60 min | 35.17 ± 0.4083 | 6 | 34.12 – 36.22 |
| Hydroxybutyrate | 0 min | 4.576 ± 0.9993 | 6 | 2.007 – 7.145 |
|  | 20 min | 59.55 ± 1.030 | 6 | 56.90 – 62-20 |
|  | 60 min | 80.04 ± 0.4568 | 6 | 78.87 – 81.22 |
| Citrate | 0 min | 0.9750 ± 0.09758 | 6 | 0.7241 – 1.226 |
|  | 20 min | 5.633 ± 0.3063 | 6 | 4.845 – 6.420 |
|  | 60 min | 10.76 ± 0.2363 | 6 | 10.15 – 11.36 |
| cis-Aconitate | 0 min | 0.1082 ± 0.04438 | 6 | -0.005886 – 0.2223 |
|  | 20 min | 2.025 ± 0.2010 | 6 | 1.509 – 2.542 |
|  | 60 min | 3.698 ± 0.1842 | 6 | 3.225 – 4.171 |
| Isocitrate | 0 min | 0.5272 ± 0.07423 | 6 | 0.3363 – 0.7180 |
|  | 20 min | 3.416 ± 0.2555 | 6 | 2.759 – 4.073 |
|  | 60 min | 7.765 ± 0.5219 | 6 | 6.423 – 9.106 |

| α-Ketoglutarate | | 0 min | | 0.2138 ± 0.005152 | | 6 | 0.008134 – 0.03462 |
| --- | --- | --- | --- | --- | --- | --- | --- |
|  |  | 20 min | | 0.4388 ± 0.02331 | | 6 | 0.3789 – 0.4987 |
|  |  | 60 min | | 1.026 ± 0.06081 | | 6 | 0.8697 – 1.182 |
| Succinyl-CoA | | 0 min | | 0.05686 ± 0.05686 | | 6 | -0.08930 – 0.2030 |
|  |  | 20 min | | 0 ± 0 | | 6 | 0 – 0 |
|  |  | 60 min | | 0 ± 0 | | 4 | 0 – 0 |
| Succinate | | 0 min | | 0.04172 ± 0.008589 | | 6 | 0.01964 – 0.06380 |
|  |  | 20 min | | 0.3410 ± 0.01696 | | 6 | 0.2974 – 0.3846 |
|  |  | 60 min | | 0.8723 ± 0.04888 | | 6 | 0.7467 – 0.9980 |
| Fumarate | | 0 min | | 0.03026 ± 0.007521 | | 6 | 0.01093 – 0.04959 |
|  |  | 20 min | | 1.956 ± 0.1039 | | 6 | 1.689 – 2.223 |
|  |  | 60 min | | 3.984 ± 0.3929 | | 6 | 2.974 – 4.994 |
| Malate | | 0 min | | 0.07637 ± 0.008878 | | 6 | 0.05355 – 0.09920 |
|  |  | 20 min | | 2.013 ± 0.09125 | | 6 | 1.778 – 2.247 |
|  |  | 60 min | | 2.983 ± 0.1951 | | 6 | 2.482 – 3.485 |
| **Metabolite** | **Comparison** | | **Adjusted p-value** | | **One-way ANOVA, Tukey´s multiple comparison test** | | |
| Butyric acid | 0 min – 20 min | | 0.0003 (***) | | P value < 0.0001; R squared 0.7633 | | |
|  | 0 min – 60 min | | < 0.0001 (****) | |  |  |  |
|  | 20 min – 60 min | | 0.4047 (ns) | |  |  |  |
| Butyryl-CoA | 0 min – 20 min | | 0.9984 (ns) | | P value 0.8794; R squared 0.01699 | | |
|  | 0 min – 60 min | | 0.8885 (ns) | |  |  |  |
|  | 20 min – 60 min | | 0.9113 (ns) | |  |  |  |
| 3-Hydroxybutyryl-CoA | 0 min – 20 min | | 0.0127 (*) | | P value 0.0075; R squared 0.4789 | | |
|  | 0 min – 60 min | | 0.0179 (*) | |  |  |  |
|  | 20 min – 60 min | | 0.9837 (ns) | |  |  |  |
| Acetyl-CoA | 0 min – 20 min | | < 0.0001 (****) | | P value < 0.0001; R squared 0.9953 | | |
|  | 0 min – 60 min | | < 0.0001 (****) | |  |  |  |
|  | 20 min – 60 min | | < 0.0001 (****) | |  |  |  |
| Hydroxybutyrate | 0 min – 20 min | | < 0.0001 (****) | | P value < 0.0001; R squared 0.9963 | | |
|  | 0 min – 60 min | | < 0.0001 (****) | |  |  |  |
|  | 20 min – 60 min | | < 0.0001 (****) | |  |  |  |
| Citrate | 0 min – 20 min | | < 0.0001 (****) | | P value < 0.0001; R squared 0.9836 | | |
|  | 0 min – 60 min | | < 0.0001 (****) | |  |  |  |
|  | 20 min – 60 min | | < 0.0001 (****) | |  |  |  |
| cis-Aconitate | 0 min – 20 min | | < 0.0001 (****) | | P value < 0.0001; R squared 0.9442 | | |
|  | 0 min – 60 min | | < 0.0001 (****) | |  |  |  |
|  | 20 min – 60 min | | < 0.0001 (****) | |  |  |  |
| Isocitrate | 0 min – 20 min | | < 0.0001 (****) | | P value < 0.0001; R squared 0.9393 | | |
|  | 0 min – 60 min | | < 0.0001 (****) | |  |  |  |
|  | 20 min – 60 min | | < 0.0001 (****) | |  |  |  |
| α-Ketoglutarate | 0 min – 20 min | | < 0.0001 (****) | | P value < 0.0001; R squared 0.9598 | | |
|  | 0 min – 60 min | | < 0.0001 (****) | |  |  |  |
|  | 20 min – 60 min | | < 0.0001 (****) | |  |  |  |
| Succinyl-CoA | 0 min – 20 min | | 0.5076 (ns) | | P value 0.4651; R squared 0.1111 | | |
|  | 0 min – 60 min | | 0.5779 (ns) | |  |  |  |
|  | 20 min – 60 min | | > 0.9999 (ns) | |  |  |  |
| Succinate | 0 min – 20 min | | < 0.0001 (****) | | P value < 0.0001; R squared 0.9626 | | |
|  | 0 min – 60 min | | < 0.0001 (****) | |  |  |  |
|  | 20 min – 60 min | | < 0.0001 (****) | |  |  |  |
| Fumarate | 0 min – 20 min | | < 0.0001 (****) | | P value < 0.0001; R squared 0.9044 | | |
|  | 0 min – 60 min | | < 0.0001 (****) | |  |  |  |
|  | 20 min – 60 min | | < 0.0001 (****) | |  |  |  |
| Malate | 0 min – 20 min | | < 0.0001 (****) | | P value < 0.0001; R squared 0.9496 | | |
|  | 0 min – 60 min | | < 0.0001 (****) | |  |  |  |
|  | 20 min – 60 min | | 0.0002 (***) | |  |  |  |

**Supplementary Table 9: To Supplementary Figure 6**

| **Figure** | **Condition** | **Mean ± SEM** | | **n** | | **CI 95% of mean** |
| --- | --- | --- | --- | --- | --- | --- |
|  | no flies 0 min | 596497 ± 18868 | | 3 | | 515313 – 677680 |
|  | no flies 20 min | 624007 ± 59654 | | 4 | | 434161 – 813853 |
|  | no flies 60 min | 589209 ± 5924 | | 4 | | 570357 – 608062 |
|  | flies 20 min | 678336 ± 16430 | | 3 | | 607643 – 749028 |
|  | flies 60 min | 740206 ± 126452 | | 3 | | 196127 – 1284285 |
|  | **Comparison** | | **Adjusted p-value** | | **One-way ANOVA, Tukey´s multiple comparison test** | |
|  | no flies 20 min – flies 20 min | | 0.9630 (ns) | | P value 0.4080; R squared 0.2652 | |
|  | no flies 20 min – no flies 60 min | | 0.9903 (ns) | |  |  |
|  | no flies 20 min – flies 60 min | | 0.6422 (ns) | |  |  |
|  | no flies 20 min – no flies 0 min | | 0.9971 (ns) | |  |  |
|  | flies 20 min – no flies 60 min | | 0.8183 (ns) | |  |  |
|  | flies 20 min – flies 60 min | | 0.9538 (ns) | |  |  |
|  | flies 20 min – no flies 0 min | | 0.8843 (ns) | |  |  |
|  | no flies 60 min – flies 60 min | | 0.4104 (ns) | |  |  |
|  | no flies 60 min – no flies 0 min | | >0.9999 (ns) | |  |  |
|  | flies 60 min – no flies 0 min | | 0.5167 (ns) | |  |  |
